# The ethical challenges of artificial intelligence‐driven digital pathology

**DOI:** 10.1002/cjp2.263

**Published:** 2022-02-17

**Authors:** Francis McKay, Bethany J Williams, Graham Prestwich, Daljeet Bansal, Nina Hallowell, Darren Treanor

**Affiliations:** ^1^ The Ethox Centre and the Wellcome Centre for Ethics and Humanities, Nuffield Department of Population Health University of Oxford Oxford UK; ^2^ Department of Histopathology Leeds Teaching Hospitals NHS Trust Leeds UK; ^3^ Department of Pathology University of Leeds Leeds UK; ^4^ Patient and Public Engagement Lead Yorkshire and Humber Academic Health Science Network Wakefield UK; ^5^ Department of Clinical Pathology Linköping University Linköping Sweden; ^6^ Department of Clinical and Experimental Medicine Linköping University Linköping Sweden; ^7^ Center for Medical Image Science and Visualization (CMIV) Linköping University Linköping Sweden

**Keywords:** artificial intelligence, digital pathology, ethics, privacy, autonomy, choice, equity, bias, commercialisation, trust

## Abstract

Digital pathology – the digitalisation of clinical histopathology services through the scanning and storage of pathology slides – has opened up new possibilities for health care in recent years, particularly in the opportunities it brings for artificial intelligence (AI)‐driven research. Recognising, however, that there is little scholarly debate on the ethics of digital pathology when used for AI research, this paper summarises what it sees as four key ethical issues to consider when deploying AI infrastructures in pathology, namely, privacy, choice, equity, and trust. The themes are inspired from the authors' experience grappling with the challenge of deploying an ethical digital pathology infrastructure to support AI research as part of the National Pathology Imaging Cooperative (NPIC), a collaborative of universities, hospital trusts, and industry partners largely located across the North of England. Though focusing on the UK case, internationally, few pathology departments have gone fully digital, and so the themes developed here offer a heuristic for ethical reflection for other departments currently making a similar transition or planning to do so in the future. We conclude by promoting the need for robust public governance mechanisms in AI‐driven digital pathology.

## Introduction

In 2018, the UK Government invested £50 million to establish five artificial intelligence (AI) Centres of Excellence in digital pathology and medical imaging, as part of the ‘data to early diagnosis and precision medicine’ strand of their Industrial Strategy Challenge Fund. The five centres are the Industrial Centre for AI Research in Digital Diagnostics (I‐CAIRD), the London Medical Imaging and Artificial Intelligence Centre for Value‐Based Healthcare, the National Consortium of Intelligent Medical Imaging (NCIMI), the National (previously Northern) Pathology Imaging Collaborative (NPIC), and the Pathology Image Data Lake for Analytics, Knowledge and Education (PathLAKE). This paper examines the ethical challenges arising in the delivery of one side of that investment, namely, digital pathology.

Digital pathology – the digitalisation of clinical histopathology services through the scanning and electronic storage of pathology slides – has opened up new possibilities for health care in recent years, including improved efficiency and safety, remote working, and reduced costs [1, 2]. Most notable, however, are the opportunities it brings for AI‐driven research. Yet, although there is growing literature on the ethics of medical AI and imaging [3, 4, 5], there is, as Coulter *et al* have noted, a lack of understanding amongst histopathologists regarding the ethics of AI‐driven digital pathology [6]. The purpose of this paper is therefore to provide a timely intervention into digital pathology research ethics by reviewing key issues to consider when deploying AI infrastructures.

Here, we focus on four themes: privacy, choice, equity, and trust. The themes are not meant to be exhaustive, but instead are general introductory frameworks meant to stimulate debate and reflection. As Morley *et al* note, ethical issues are relative to ‘different stages of the algorithmic development lifecycle’ [5]. The issues raised here, then, will likely evolve as digital pathology develops. As, however, there are presently no AI tools being applied in routine diagnostic work in the UK, rather than speculate on the potential bioethical effects of unknown future AI tools, the paper looks instead at the current state of digital pathology practice in the UK, and the ethical challenges arising out of the attempt to provide an infrastructure for AI research in digital pathology.

The themes are inspired from our experience of grappling with the challenge of deploying an ethical digital pathology infrastructure to support AI research as part of the National Pathology Imaging Cooperative (NPIC), a collaborative of universities, hospital trusts, and industry partners largely located across the North of England. Though focusing on the UK case, internationally, few pathology departments have gone fully digital, and so the themes developed here offer a heuristic for ethical reflection for other departments currently making a similar transition or planning to do so in the future. The paper begins with a description of the data sharing practices and privacy protections already in place within NPIC. It uses that as a springboard for discussing ethical issues we have encountered when thinking about data sharing beyond the clinical care context, namely, those of choice, equity, and trust. Following the outlining of those issues, we conclude by arguing for the need for robust public governance frameworks to strengthen accountability over the sharing of digital pathology data when developing medical AI tools.

## Privacy

**Figure 1:**
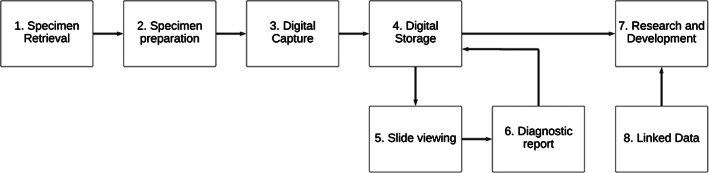
A simplified model of digital pathology work and data flows. The process begins with specimen retrieval (1) and preparation (2) through fixing, cut‐up, embedding, microtomy, staining, and drying. High‐resolution digital scans (×40) are then taken and stitched into one ‘WSI’ (3). The image is then uploaded to a server (4) to be viewed by the pathologist on their workstation (5) and they input a digital report (6). The pathology report data (e.g. summary diagnosis, free text, and the minimum pathology data set required by COSD [Cancer Outcomes and Services Dataset]) are stored on the server along with the patient's histopathology image data as identifiable information. It is then de‐identified upon export for the purposes of research (7) and made accessible along with other linked data (8).

Traditionally, histopathologists have diagnosed disease by examining sections of tissue on glass slides with a light microscope. With the introduction of whole slide image (WSI) scanners, however, glass slides can be captured as high‐resolution digital images, stored and transmitted electronically, and viewed on workstations allowing pathologists to make their diagnosis using an enlarged digital image on their computer screen. Databases of the captured images can also be used to train machine learning algorithms. Figure 1 provides a simplified model of digital pathology work and data flows.

The process begins with specimen retrieval and preparation through fixing, cut‐up/trimming/grossing, embedding, microtomy, staining, and drying. High‐resolution digital scans (×40) of the glass slide are then taken and ‘stitched’ into one ‘WSI’. Next, the digital images are uploaded to a server and viewed by the pathologist on their workstation so that they can determine their diagnosis and input a digital report. The pathologist may also, at this point, add annotations and labels to the images as part of their reporting. That information is saved with the image and report, and stored in the cloud where it can be accessed, along with other linked data, by third parties for the purposes of research.

Though there are several ethical issues that may arise throughout the workflow, the ethical challenges related to AI are primarily associated with the pooling and sharing of data for research (research here may involve multiple uses of patient data, whether AI driven or not, but it is expected that AI will be the most common contribution due to its facility in analysing large data sets). Here, the issue that has seen the most attention is data privacy and its protection through de‐identification practices.

The common law duty of confidentiality holds that a doctor has an obligation to keep identifiable patient information – i.e. information that might disclose a patient's identity beyond the direct care team – private, unless given explicit consent to share it, or sharing it falls under one of the exceptions to the common law duty: anonymity, legal obligation, public interest, impracticality, etc. [7]. Histopathology has traditionally maintained privacy through physical limitations of glass slides sharing (which are bound to one site at any one time) and through removing or obscuring patient identifiers (for instance, removing labels or scrubbing out information using black out marker pens). Digital pathology data are duplicable, more mobile, and hence require additional techniques to ensure the privacy of patient data.

Data privacy can be assured in several ways, for instance, by managing access to the data, through de‐identification techniques (i.e. by removing data that could identify a patient), and by legally binding third parties to maintaining secure information governance practices. In NPIC, we achieve this by controlling access to data through the Leeds Teaching Hospital Trust data access committee, who review researcher applications according to, amongst other things, the scientific merit of the research plan, the benefits to patient communities, and conformity with data protection law [8]. Upon successful application, the data extraction team de‐identify data by suppressing, randomising, or generalising direct and indirect identifiers such as NHS numbers, dates of birth, dates of admission, etc. In doing so, they use ‘K‐anonymity’ methods in which data are de‐identified to be indistinguishable from a set amount (*k*) of other individuals. Moreover, as studies show that linking data sets can theoretically increase the possibility of re‐identification [9], there are further safeguards in place to limit the risks to privacy. This includes secure transfer and storage mechanisms, data destruction policies, audit trails recording what data were released and to whom, and legal safeguards in the form of contracts forbidding any attempt to re‐identify patients.

In many ways, the above data protection measures build upon existing practices for medical data sharing in hospital settings in the UK, and collectively they provide a robust system for protecting patient data when sharing it for research. It is a primary obligation for any department hoping to deploy digital pathology systems for AI research to ensure this continues to be the case and to commit to ongoing monitoring of data protection measures to make sure they remain up to date.

## Choice

Even with safeguards in place for protecting patient privacy, as a report from Understanding Patient Data and the Ada Lovelace Institute put it, ‘people may still care how…[data] is used even if they can't easily be identified’ [10]. For instance, they may care about how such data are used because of concerns over the potential social harms stemming from its use and thus may wish to exercise autonomy over who can access the data and the uses to which it is put. Hence, the question arises: what choice should the public have over the sharing of their de‐identified pathology data? One way of thinking about that question of autonomy over data sharing is through the issue of consent.

It is a well‐established principle of medical law and ethics that patients provide informed consent for their treatment [11]. Individual opt‐in consent is, as noted above, also required when sharing personal data unless sharing it falls under one of the aforementioned exceptions or the implied consent needed for direct care. Processing digital slides in the hospital trust all fall under data practices needed for direct care. When it comes to sharing pathology data for research purposes, however, no standard process exists. For instance, with NPIC‐controlled data, some external researchers wanting access to the data may voluntarily seek explicit opt‐in consent from patients as part of their ethics approvals before applying to our data access committee, while certain forms of linked data may already have arrangements for explicit consent for sharing data for research (for instance, the 100,000 Genomes Project, which is to be linked to the NPIC data set, has explicit opt‐in consent as a requirement) [12]. Otherwise, however, explicit opt‐in consent is not a requirement, following recommendations by the General Medical Council (GMC) for sharing de‐identified pathology images [13]. The ethical grounds for sharing pathology images without consent are found in the anonymity of the data, which is one of the exceptions to the common law duty of confidentiality [14]. Though anonymity in its strongest sense means having no possible way to link back to any information that may identify someone, here anonymity has a more practical meaning of low‐risk identifiability, and thus is synonymous with robust de‐identification. Such an interpretation is backed up by the Information Commissioners Office (ICO), who confirm that the anonymity exception applies even if there are limited risks of re‐identification [9]. Similarly, the Data Protection Act (2018) and case law both confirm that anonymisation does not have to be ‘risk free’, only that the risks of identification be mitigated so as to be remote or unlikely. Even where the data controller holds information that may allow for the possibility of re‐identification, that in itself is not sufficient grounds for constituting the disclosure of personal information [9]. Though recognising that anonymity is potentially problematic in an era of big data, there is no *a priori* reason to think, then, that sharing de‐identified digital pathology data along with linked data would fail a test of low‐risk anonymity.

De‐identification of patient data informs NPIC's approach to consent, which is to share de‐identified data without individual consent so as to maximise the utility of healthcare data for public benefit. We believe this aligns with public opinion, insofar as the public generally are more willing to share data when de‐identified [15]. We also recognise, however, that patients may want to exercise some control over data flows, even when de‐identified, which we achieve by checking against national opt‐outs before data release. The decision to follow the national opt‐out is voluntary, since it is only mandatory when sharing identifiable information without consent (i.e. when sharing falls under section 251 of the NHS Act 2006, which allows confidentiality to be set aside for identifiable information under certain conditions) [16]. It does not apply, therefore, for de‐identified data, such as that used in NPIC.

Though opt‐in consent (whether broad, dynamic, or meta) might offer alternatives for enhancing autonomy for data subjects, it can be difficult to administer at scale or for retrospective data, and would likely also reduce participation rates, compromising the utility of the database as a result [17, 18]. Opt‐out models are arguably easier to implement, though we recognise that there are risks with these models as well. For instance, it is questionable whether opt‐outs provide genuine choice if patients are unaware of that option and it is possible that they can introduce bias into the data set if certain groups of people opt‐out [19]. Robust public engagement would limit the risk of the former. Regarding the latter, it is not known what the specific risks are for digital pathology, though there are reasons to be optimistic. For instance, the latest figures for the national data opt‐out (for September 2021) show an opt‐out rate of 5.35% [20]. According to one study, this is within the tolerances of a meaningfully representative study of population health, which puts participation requirements at 90% [21]. That said, biases remain a *theoretical* possibility if systematic opt‐outs occur within that margin (and especially if the margin grows).

These risks notwithstanding, following the national opt‐out, we argue, is a reasonable and practical alternative to opt‐in measures, providing patients with autonomy around sharing de‐identified data, while balancing that against the need for maximising data sharing and protecting patient confidentiality. As with privacy, we also advise that opt‐out risks should be continually monitored and mitigated where possible.

## Equity

Given the low risks to patient confidentiality, the public benefits of sharing data, and the availability of opt‐out, we contend that the above approach functions well for protecting individual data subjects' interests for privacy and autonomy. However, there are broader social challenges of AI‐driven digital pathology concerning equity that need to be mentioned.

For instance, one of the most pressing challenges that comes up in medical AI literature is that of data value and for‐profit involvement. As several studies confirm, the public are generally supportive of sharing NHS data for purposes beyond direct care, but that support is largely conditional on there being public, rather than merely private, benefits [22, 23]. This does not mean that for‐profit motives cannot play any role whatsoever. Evidence also suggests that the public are increasingly aware of the value of NHS data, and are also of the opinion that its value can be returned in direct (health care) and indirect (financial, administrative, etc.) benefits [10, 24]. Notwithstanding this broad understanding of reciprocity, evidence shows that there is common scepticism over commercial data sharing agreements, especially in instances where commercial partners bargain for sharing agreements that are disproportionately favourable to their private interests over public benefit [10, 22, 25, 26].

Given that large technology and social media companies, who possess the advanced engineering expertise and technical resources for supporting AI research at scale, play an important role in (AI driven) healthcare research, the question becomes how to offer a balanced and fair model of data sharing practices with researchers, including those working for profit? Contemporary responses to this question have been to offer guidance on best practices through, for instance, checklists for AI procurement, financial compensation schemes, and promises of an NHS centre of expertise to provide guidance on data value [27, 28]. Such advice, however, may limit the perceived value of data to financial value and therefore raises further questions about how to diversify what constitutes value and fair reciprocity.

Another challenge for healthcare equity comes from the possibility of algorithmic bias. Although algorithmic bias has primarily been observed outside of healthcare contexts, it has recently been shown to have implications for patient communities as well [29, 30, 31]. As Hao notes [32], there are different ways in which bias can find its way into AI, from underrepresented data sets to poor conceptualisation of research. It is unclear what pathways exist for bias in AI‐driven digital pathology. On one hand, digital pathology cohort identification strategies build upon existing approaches to cohort selection in pathology and clinical trials, meaning any possible bias would not necessarily be an outcome of digital pathology *per se*, but of those existing practices. There is also a widespread assumption that common cancer cells exhibit a morphological uniformity across cohorts, implying that the risks of bias are minimal. On the other hand, some cancers are understood to disproportionately affect (whether due to prevalence or aggressiveness) some groups more than others [33, 34], or are simply rarer as in the case of sarcomas, and so may introduce biases if underrepresented in the training data. Further research is needed on the pathways in which algorithmic bias may be possible. Taken with the public concerns around commercialisation, it prompts ongoing reflection on how to make medical AI research equitable and available to all.

## Trust

Both commercial involvement and bias have further implications for public trust. Kerasidou defines trust in terms of three core concepts: vulnerability, voluntariness, and good will [35]. Thus, for trust to exist, an individual or organisation makes decisions that are in the best interests of another, whose trust must be earned, not obliged. Trustworthiness similarly means being able to demonstrate that goodwill. Though there are no studies of public trust in AI‐driven digital pathology, multiple studies show differing degrees of awareness and trust around AI. A recent public survey conducted for the British Computer Society highlights that approximately half (53%) of 2,000 adults surveyed claimed they had ‘no faith in any organisation to use algorithms when making judgements about them’ [36]. Only 17% of respondents trusted the uses of automated decisions in the NHS. Research conducted by Ipsos Mori is more optimistic. Overall support for data‐driven technologies, especially those using scans or imaging for the automation of diagnosis, was high, providing that humans were kept in the loop [37, 38].

One area where trust is most fragile, however, is in the involvement of commercial third parties, especially social media, insurance, and marketing companies, and those based in the USA, which are consistently rated as having the highest degrees of mistrust [39]. Insofar as those companies may want access to digital pathology data for research, even if the public are amenable to AI data analysis for diagnosis, they may not trust the company applying to make use of their data, and therefore desire assurances that any commercial involvement is overall in the public interest [35]. Similarly, there is much documented mistrust affecting the acceptance of, or participation in, AI or health research, in minority or marginalised communities, often due to histories of bias or exclusion [40, 41, 42].

Mistrust may also be exacerbated by the media. For instance, it has been noted that there are pervasive anxieties regarding AI stemming from the perceived anticipatory harms and sometimes apocalyptic imagery referenced in works of fiction [43]. Understanding Patient Data have also highlighted how news media often focuses on the risks of health data sharing rather than its benefits [44]. Arguably, these media portrayals of the risks of AI are likely to impact perceptions of the trustworthiness of future medical AI, and taken with the mistrust due to commercialisation and bias, emphasise the need to develop medical AI systems that are worthy of trust.

Trustworthiness has been shown to be earned in a number of ways in medical contexts, for instance, through consent processes, professional integrity, and community engagement [35]. In the context of AI, it is argued that trustworthiness is earned by developing algorithms that are lawful, ethical, and robust and which respect principles of human autonomy, prevention of harm, fairness, and explicability (the last of which includes ideals of transparency and explainability) [45, 46]. All of these norms will no doubt continue to be important to the public acceptability of AI. That said, there is some controversy around the notion of explainability. Though a common aspiration for the development of ethical AI [47], Ghassemi *et al* [48] have recently called it a ‘false hope’ due to interpretability gaps between AI models and current explainability methods. Instead, they advocate for rigorous internal and external validation of AI tools in order to engender trust. Validation will no doubt be an essential mechanism for promoting public confidence, and, notwithstanding Ghassemi *et al*'s point, how AI is transparently communicated to the public should also remain a vitally important issue for the future ethics of medical AI research [49].

## Conclusion

Privacy, choice, equity, and trust do not exhaust the ethical issues emerging in AI‐driven digital pathology, but they are central to the use of data for research purposes in the field. As it stands, medical data sharing ethics tend to focus on issues of privacy in order to protect individual research subjects' interests. When thinking about the ethical challenges of sharing pathology data, however, a broader vantage point is needed, meaning that digital pathologists also need to strengthen commitments to choice, equity, and trust. Though the issues are complicated, requiring ongoing discussion within the research community, it is also important to remember the role patient and public involvement and engagement (PPIE) has in all this. As a recent survey of histopathologists in the UK confirms, there is a general ‘need for transparency about data uses and the inclusion of the views and opinions of the public in decisions about these uses’ [6]. One reason, however, why medical data sharing practices sometimes struggle stems from a failure to properly include patients and the public in the governance process [50, 51]. To strengthen commitments to privacy, choice, equity, and trust, then, it would be wise to learn from that, and to involve the public in those discussions with researchers and ethicists as they think through the future of medical AI.

## Author contributions statement

All authors were involved in theorising and writing the paper and had final approval of the submitted and published versions.
